# In vitro feeding of all life stages of two-host *Hyalomma excavatum* and* Hyalomma scupense* and three-host *Hyalomma dromedarii* ticks

**DOI:** 10.1038/s41598-023-51052-w

**Published:** 2024-01-03

**Authors:** Khawla Elati, Hayet Benyedem, Kohsuke Fukatsu, Peggy Hoffmann-Köhler, Moez Mhadhbi, Serkan Bakırcı, Hüseyin Bilgin Bilgiç, Tülin Karagenç, Mohamed Aziz Darghouth, Ard M. Nijhof

**Affiliations:** 1 Institute of Parasitology and Tropical Veterinary Medicine, Freie Universität Berlin, Robert-Von-Ostertag-Str. 7, 14163 Berlin, Germany; 2 Veterinary Centre for Resistance Research, Freie Universität Berlin, Robert-Von-Ostertag-Str. 8, 14163 Berlin, Germany; 3 Laboratoire de Parasitologie, École Nationale de MédecineVétérinaire de SidiThabet, Institution de la Recherche et de l’Enseignement Supérieur Agricoles, Univ. Manouba, 2020 Sidi Thabet, Tunisia, Univ. Manouba, Sidi Thabet, Tunisia; 4 Research Center, Nihon Nohyaku Co., Ltd., Osaka, Japan; 5 Faculty of Veterinary Medicine, Department of Parasitology, Aydın Adnan Menderes University, Isıklı‑Efeler, Aydın, Turkey

**Keywords:** Biological techniques, Biotechnology

## Abstract

Ticks are blood-sucking ectoparasites and can transmit various pathogens of medical and veterinary relevance. The life cycle of ticks can be completed under laboratory conditions on experimental animals, but the artificial feeding of ticks has attracted increased interest as an alternative method. This study represents the first report on the successful in vitro feeding of all life stages of two-host tick species, *Hyalomma scupense* and *Hyalomma excavatum,* and the three-host tick *Hyalomma dromedarii*. The attachment and engorgement rates of adults were 84% (21/25) and 76% (19/25) for *H. scupense* females. For adult *H. excavatum* and *H. dromedarii*, 70% (21/30) and 34.4% (11/32) of the females attached and all attached females successfully fed to repletion. The oviposition rates of the artificially fed females were 36.4%, 57.1% and 63.1% for *H. dromedarii, H. excavatum* and *H. scupense*, respectively, with a reproductive efficiency index varying between 44.3 and 60.7%. For the larvae, the attachment and engorgement rates were 44.2% (313/708) and 42.8% (303/708) for *H. dromedarii*, 70.5% (129/183) and 56.8% (104/183) for *H. excavatum* and 92.6% (113/122) and 55.7% (68/122) for *H. scupense*. The attachment and engorgement rates for the nymphs were 90.2% (129/143) and 47.6% (68/143) for *H. dromedarii*, 66.7% (34/51) and 41.2% (21/51) for *H. excavatum*, and 44.1% (30/68) and 36.8% (25/68) for *H. scupense*. Molting rates of the immature stages varied between 71.3% (216/303) and 100% (68/68) for the larvae and between 61.9% (13/21) and 96% (24/25) for the nymphs. The successful in vitro feeding of all stages of the three *Hyalomma* species makes this method a valuable tool for tick research, with potential applications in studies on the pathogens transmitted by these tick species such as *Theileria annulata*.

## Introduction

Ticks are hematophagous ectoparasites that may act as vectors for a variety of pathogens infecting humans and animals worldwide. This includes several species of the *Hyalomma* genus that are associated with the transmission of Crimean-Congo Hemorrhagic Fever virus in humans^1,2^ and *Theileria annulata*, the causal agent of tropical theileriosis in livestock^3^.

The duration of tick life cycle is strongly affected by environmental factors, e.g. temperature and relative humidity and by the availability of hosts^4^. *Hyalomma scupense*, *H. dromedarii* and *H. excavatum* have different life cycles. In Tunisia, *H. scupense* was found to behave as a two-host tick species with peak activity of the adults feeding on cattle in summer^5^, whereas in other regions, a cold-adapted ecotype of *H. scupense* was found to have a one-host life cycle^6^. *Hyalomma scupense* collected in China were reported to behave as one- and two-host tick under laboratory conditions^7^. *Hyalomma excavatum* can have a two-or three host life cycle tick depending on the availability of hosts. In North Africa, it is active during the whole year with a peak in spring. Larvae and nymphs of this species feed on rodents and the adults infest various hosts such as cattle, small ruminants and equids^8^. *Hyalomma dromedarii* can behave as a one-, two-, or three-host tick^8–10^, whereby the immature stages can feed on camels, but also on rodents and birds. The activity of this ticks species does not exhibit a seasonal variation and it can be found throughout the year on animals^11,12^.

Studies on the biology of these ticks frequently require laboratory tick colonies that are reared and maintained on animals^29^. In recent years, artificial tick feeding systems (ATFS) have been developed and adapted for different ixodid ticks such as *Amblyomma hebraeum*, *Dermacentor reticulatus, Ixodes ricinus, Rhipicephalus appendiculatus* as well as nymphs and adult *Hyalomma* species such as *H. dromedarii* and *H. anatolicum*^13–19^. The development and optimization of methods to feed hard ticks artificially in the laboratory not only contributes to the 3Rs principle (to Reduce, Replace and Refine animal experiments) for humane animal research^20^, but also provides researchers with a versatile tool to study various aspects of tick biology, pathogen transmission and drug discovery under controlled laboratory conditions^13,21^.

However, extensive optimization of the artificial feeding method is still required. There is for instance a lack of studies examining the artificial feeding of juvenile stages of one- and two-host tick species where ticks molt on the host to the subsequent life stage. In addition, the success rate reported for the in vitro feeding of ixodid ticks varies considerably and the addition of antibiotics to the blood meal to prevent microbial contamination may exert a negative effect on tick endosymbionts and tick fecundity^22,23^. In this study, we targeted the first point by feeding all life stages of *H. dromedarii, H. excavatum* and *H. scupense* in vitro.

## Results

### Feeding of *Hyalomma* adults

#### In vitro feeding

**Figure 1:**
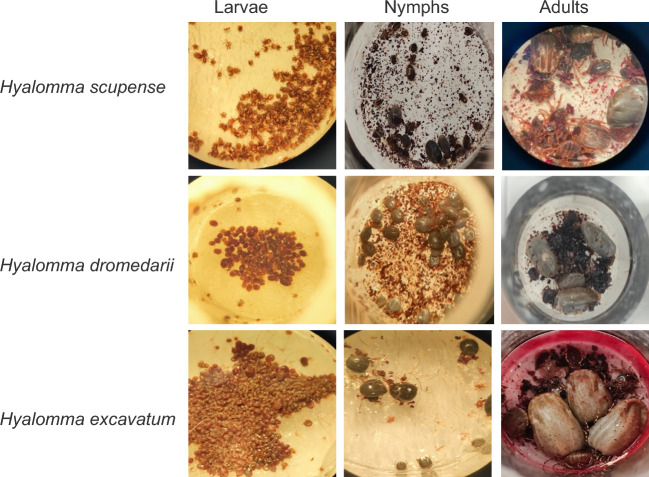
Attachment and engorgement of different *Hyalomma* life stages in vitro.

All life stages of *H. excavatum*, *H. dromedarii* and *H. scupense* were successfully fed in vitro (Fig. 1). Results of the in vitro feeding of adult *Hyalomma* ticks are summarized in Table 1. Briefly, *H. excavatum* females (fed in winter, January) achieved a 70% (21/30) attachment rate where all of the attached ticks (21/21) engorged. The mean weight of engorged females was 539.8 ± 163 mg. Twelve of the 21 engorged females (57.1%) laid eggs (6665 ± 2009 eggs), with a mean REI of 51.8 ± 11.9%, where 11 of the females (91.6%) produced viable larvae. The percentage of hatched larvae varied between 90 and 100% (except for one tick where the hatching rate was 30%) resulting in a mean of 6217 ± 1031 larvae (Table 1).

**Table 1:** In vitro feeding of adult *Hyalomma* species.

	*H. dromedarii*	*H. excavatum*	*H. scupense W*	*H. scupense S*
Attachment rate (%)	11/32 (34.4%)	21/30 (70%)	18/20 (90%)	21/25 (84%)
Engorgement rate (%)	11/32 (34.4%)	21/30 (70%)	5/20 (25%)	19/25 (76%)
Mean feeding duration (days ± SD, [range])	9 ± 1.7 [6–12]	7.6 ± 1.6 [6–10]	9.2 ± 2.5 [7–12]	11.5 ± 2.2 [7–14]
Mean detachment weight (mg ± SD, [range])	276 ± 105 [67–448]	539.8 ± 163 [200–768]	36.8 ± 22.5 [16–63.3]	298 ± 65 [160–378]
Oviposition rate (%)	4/11 (36.4%)	12/21 (57.1%)	0	12/19 (63.1%)
Mean duration of pre-oviposition (days ± SD, [range])	12.6 ± 2.5 [10–15]	58.2 ± 29 [20–96]	–	7.2 ± 1.3 [6–10]
Mean egg mass (mg)	204.25 ± 150 [1–338]	337.3 ± 101 [223–508]	–	134.4 ± 45 [58–213.5]
Mean reproduction efficiency index (%) (Mean ± SD [range])	60.7 ± 40 [0.2–83]	51.8 ± 11.9 [35–68]		44.3 ± 7.3 [29–59]
Mean number of eggs produced ± SD [range]	4036 ± 2967 [20–6679]	6665 ± 2009 [4406–10,038]	–	2656 ± 902 [1146–4218]
Mean oviposition-hatching (days ± SD, [range])	39.7 ± 13 [30–59]	34.4 ± 14 [15–54]	–	43.11 ± 5.8 [36–55]
Female producing fertile eggs (%)	4/3 (100%)	11/12 (91.6%)	–	9/12 (75%)
Mean hatching rate (% ± SD, [range])	99.5 ± 0.005% [99–100%]	84.1 ± 27% [30–100%]	–	54.4 ± 39% [5–95%]
Mean number of larvae produced (± SD, [range])	4010 ± 2949 [20–6612]	6217.5 ± 1031 [5493–7706]	–	1239 ± 1036 [111–3058]

*Hyalomma dromedarii* fed in winter (January) reached an engorgement rate of 34.4% (11/32 females). The mean weight of engorged detached females was 276 ± 105 mg. Only four out of 11 females (36.4%) produced eggs, with a mean number of 4036 ± 2967 with a REI of 60%. Most of the eggs hatched successfully (99.5%) (Table 1).

As adult *H. scupense* ticks are under natural conditions in Tunisia (from where the tick colony originates) active in summer*,* we tried to examine this seasonal behaviour in vitro by conducting one feeding experiment in winter (January) and one in summer (June). This seasonal difference was not evaluated for the other two tick species as their activity does not exhibit seasonal variation. *Hyalomma scupense* adults that fed in winter showed lower engorgement rates compared to those fed in summer. Despite the high attachment rate observed in winter (18 out of 20 ticks, 90%), only five ticks (5/20, 25%) partially engorged and detached with a low mean detachment weight of 36.8 ± 22.5 mg. Whereas in summer, the attachment and engorgement rates were 84% (21/25) and 76% (19/25), respectively, with a mean detachment weight of 298.0 ± 65.4 mg. None of the females fed during the first experiment laid eggs, whereas females from the second in vitro experiment had an oviposition rate of 63.2% (12/19) and produced a mean number of eggs of 2656 ± 902 with a mean REI of 44.3 ± 7.3% (Table 1). Out of the 12 females laid eggs, nine were able to produce viable larvae (75%) with a hatching rate estimated to 54%.

#### In vivo feeding results and comparison with in vitro feeding of *Hyalomma* adults

**Figure 2:**
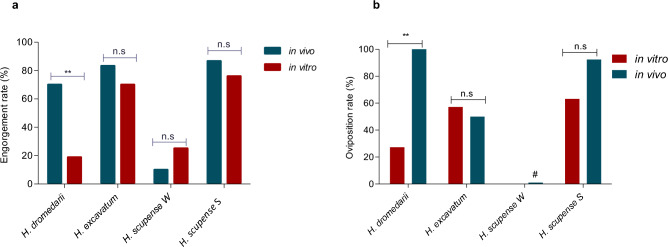
Engorgement (**a**) and oviposition (**b**) rates of adult *Hyalomma* fed in vivo and in vitro. Asterisks indicate level of significance using Z-test (*P* < 0.01 (**), n.s = non-significant, # for *Hyalomma scupense* fed in winter, only one engorged tick laid eggs and the oviposition rate was not calculated. *H. scupense* S = *Hyalomma scupense* fed in summer (S); *H. scupense* W = *Hyalomma scupense* fed in winter (W). Engorgement rate (%) = (number of engorged females/numbers of initially placed ticks) *100; Oviposition rate (%) = (number of females laying eggs/number of engorged females) *100.

The engorgement rates of *H. excavatum* and *H. dromedarii* females fed on rabbits (“in vivo”, fed in winter and in summer, respectively) were 84% (10/12) and 70% (14/20), higher than their counterparts fed in vitro (Z-test, *P* = 0.35 for *H. excavatum* and *P* = 0.01 for *H. dromedarii*). For *H. scupense* fed in winter, the engorgement rate was low for both conditions but still higher in vitro 25% (5/20) than on calf 10% (1/10) (Z-test, *P* = 0.33) (Fig. 2a), but none of the in vitro fed ticks laid eggs whereas the one female that fed to repletion on a calf did. *Hyalomma scupense* fed in summer had engorgement rates of 76% (19/25) in vitro and 86.7% (13/15) when fed on a calf (Z-test, *P* = 0.4), respectively. The oviposition rate was with 92.3% (12/13) also higher in vivo than in vitro (63.2%, 12/19; Z-test, *P* = 0.06) (Fig. 2b).

**Figure 3:**
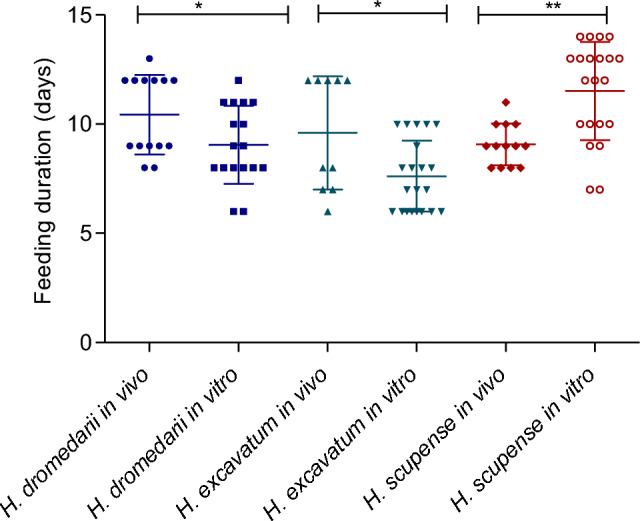
Feeding duration of engorged females *Hyalomma* fed in vivo and in vitro. Asterisks indicate level of significance using Mann Whitney test (*P* < 0.05 (*) and (*P* < 0.01 (**).

Adults of *H. dromedarii* and *H. excavatum* took longer to engorge on a rabbit (10.4 ± 1.8 days and 9.6 ± 2.5 days, respectively) than in vitro (9.1 ± 1.6 days and 7.6 ± 1.6 days, respectively) (Mann Whitney test, *P* = 0.03 for both species), whereas *H. scupense* females fed longer in vitro (11.5 ± 2.2 days) compared to *H. scupense* females fed on a calf (9.1 ± 0.95 days) (Mann Whitney test, *P* = 0.001; Fig. 3).

**Figure 4:**
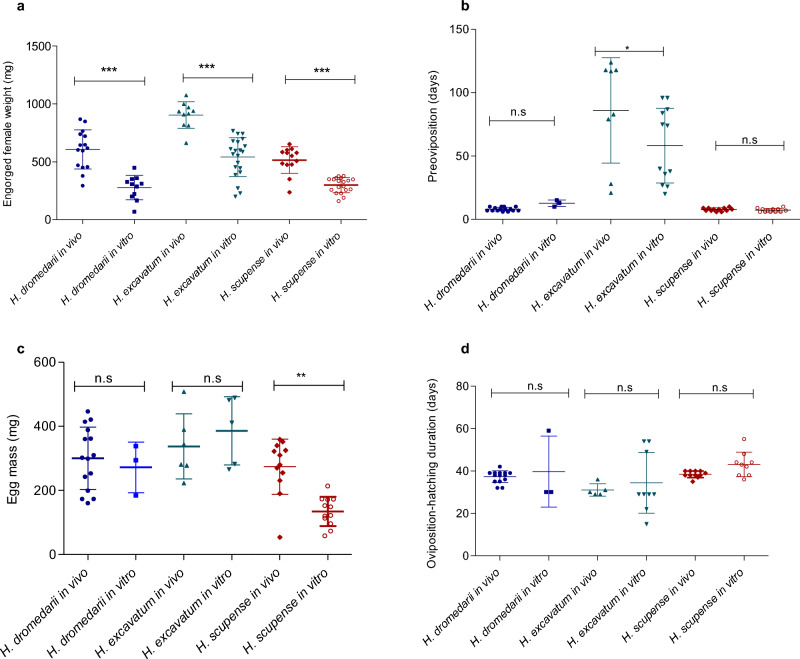
Variation of engorged female weight (**a**), duration of preoviposition (**b**), egg mass (**c**) and oviposition-hatching duration (**d**). Asterisks indicate level of significance using Mann Whitney test (*P* < 0.05 (*), (*P* < 0.01 (**), *P* < 0.0001 (***), n.s = non-significant.

The weight of engorged females was significantly higher for all *Hyalomma* ticks fed on animals compared to those fed in vitro (One-way ANOVA and Tukey test, *P* < 0.0001). The highest weight of engorged females was found for *H. excavatum* fed on an animal (903 ± 114 mg) and in vitro (540 ± 167 mg) (Fig. 4a). There were no significant differences in egg mass produced between ticks fed in vivo and in vitro for any of the species (Fig. 4c). The REI was higher in vitro than in vivo for *H. dromedarii* (60.7 and 49.5%, respectively) and *H. excavatum* (51.8 and 44.6%), but not for *H. scupense (*in vivo 52.1%, in vitro 44.3%).

The longest preoviposition period was observed for *H. excavatum* fed in vivo (86 ± 41 days) and in vitro (58 ± 29 days) with no significant difference between the two feeding conditions (Mann Whitney test, *P* = 0.1). The shortest preoviposition duration was recorded for *H. scupense* fed in vitro (7.3 ± 1.4 days) and in vivo (7.8 ± 1.2 days, Mann Whitney test, *P* = 0.2). *Hyalomma dromedarii* females fed in vivo (8 ± 1.4 days) had significantly shorter preoviposition period than *H. dromedarii* fed in vitro (12.6 ± 2.5 days) (Mann Whitney test, *P* = 0.01) (Fig. 4b).

The mean oviposition-hatching duration (the period from the start of oviposition until the hatching of the first larvae) was relatively similar between the feeding conditions (Fig. 4d).

### Feeding of larvae and nymphs

#### In vitro feeding of *Hyalomma* immatures

Results of the in vitro feeding of immature *Hyalomma* species are shown in Table 2. *Hyalomma scupense* acted as a two-host tick in vitro, i.e., the larvae molted to nymphs on the membrane (Supplementary Fig. 1) similar to ticks fed on rabbits. This contrasted with *H. dromedarii*, that acted as a three-host tick in vitro, but as a two-host tick when fed on rabbits. *Hyalomma excavatum* behaved as two-host tick when fed in vitro or on a rabbit, but as a three-host tick when the larvae were fed on a gerbil.

**Table 2:** In vitro feeding of *Hyalomma* immature stages.

	*Immature stages*	*H. dromedarii*	*H. excavatum*	*H. scupense*
Attachment rate (%)	Larvae	313/708 (44.2%)	129/183 (70.5%)	113/122 (92.6%)
	Nymphs	129/143 (90.2%)	34/51 (66.7%)	30/68 (44.1%)
Engorgement rate (%)	Larvae	303/708 (42.8%)	104/183 (56.8%)	68/122 (55.7%)
	Nymphs	68/143 (47.6%)	21/51 (41.2%)	25/68 (36.8%)
Mean feeding duration (days ± SD, [range])	Larvae	6.2 ± 1.7 [4–9]	9.3 ± 2.5 [6–13]*	16.5 ± 3.5 [14–19]*
	Nymphs	10.3 ± 2 [7–16]	7.9 ± 1 [7–13]	9.6 ± 2.6 [4–14]
Average detachment weight (mg ± SD, [range])	Larvae	NA	NA	NA
	Nymphs	12.7 ± 3.1 [6.1–19.9]	10.4 ± 2.8 [5–18.3]	13.4 ± 3.6 [6–19]
Molting rate (%)	Larvae	216/303 (71.3%)	94/104 (90.4%)	68/68 (100%)
	Nymphs	54/68 (79.4%)	13/21 (61.9%)	24/25 (96%)
Molting duration (days ± SD, [range])	Larvae	14.6 ± 2.6 [11–18]	–	–
	Nymphs	29.7 ± 7.5 [15–63]	17.9 ± 1.9 [15–20]	34.8 ± 6.1 [23–49]

*H. excavatum and *H. scupense* engorged larvae molt to nymphs while attached to the membrane, so the feeding and molting durations were given together.

A high attachment rate was observed for *H. scupense* larvae, with 113 out of 122 attached ticks (92.6%). Sixty-eight larvae out of 122 (55.7%) engorged. All engorged larvae successfully molted to the nymphal stage on the membrane. Out of these 68 nymphs, 25 fed to repletion (36.8%). Twenty-four nymphs (96%) molted to the adult stage (11 males and 13 females) in a mean of 34.8 ± 6.1 days. The mean weight of engorged nymphs was 13.4 ± 3.6 mg, whereas the mean weight of unfed adults was 6.7 ± 1.7 mg.

For *H. excavatum*, 129 out of 183 larvae attached (70.5%) and 104 engorged (56.8%). Ninety-four larvae molted (90.4%) on the membrane to the nymphal stage. An attachment rate of 66.7% (34/51), engorgement rate of 41.2% (21/51) with a mean detachment weight of 10.4 ± 2.8 mg was recorded for the nymphs. Out of 21 engorged nymphs, 13 (61.9%) molted to the adult stage (five females and eight males) in 17.9 ± 1.9 days. The average weight of unfed adults was 6 ± 2 mg.

For *H. dromedarii*, an attachment and engorgement rate of 44.2% (313/708) and 42.8% (303/708) was recorded for the larvae. Of the engorged larvae, 71.3% (216/303) molted to the nymphal stage. For the nymphs, the attachment rate was 90.2% (129/143) and the engorgement rate was 47.6% (68/143). The nymphal weight varied between 6.1 and 19.9 mg. Fifty-four (79.4%) of the nymphs molted to adults (22 females and 32 males) in a mean of 29.7 ± 7.5 days (Table 2). The attachment, engorgement and molting proportions varied significantly between the three species for larvae and nymphs (Chi-square test, *P* < 0.0001).

#### In vivo feeding results and comparison with in vitro feeding of immatures of *Hyalomma* ticks

During the in vitro feeding, *H. excavatum* and *H. scupense* behaved as two-host ticks and *H. dromedarii* acted as a three-host tick. When fed on rabbits, all three *Hyalomma* species acted as two-host ticks. *Hyalomma excavatum* acted as a three-host when larvae were fed on gerbils.

**Figure 5:**
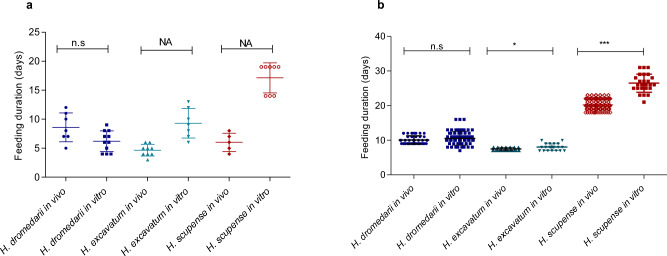
Mean feeding duration of *Hyalomma* larvae (**a**) and nymphs (**b**) fed in vitro and in vivo. NA: For *Hyalomma excavatum* and *H. scupense* larvae, statistics between in vivo and in vitro feeding condition was not conducted as ticks acted different (*H. excavatum* and *H. scupense* engorged larvae molt to nymphs while attached to the membrane, so the feeding and molting durations were given together), therefore we indicated as not available (NA). Asterisks indicate level of significance using Mann Whitney test (*P* < 0.05 (*), *P* < 0.0001 (***), n.s = non-significant.

Due to the different feeding behaviour, the only possible comparison that could be done was on the duration of feeding for *H. dromedarii* larvae*,* which was not significantly different between both conditions (Mann Whitney test, *P* = 0.06) (Fig. 5a). For the nymphs, the feeding duration varied significantly between the two feeding conditions for *H. excavatum* (Mann Whitney test, *P* < 0.01) and *H. scupense* (Mann Whitney test, *P* < 0.0001), but no significant difference was observed for *H. dromedarii* (Mann Whitney test, *P* = 0.2) (Fig. 5b).

**Figure 6:**
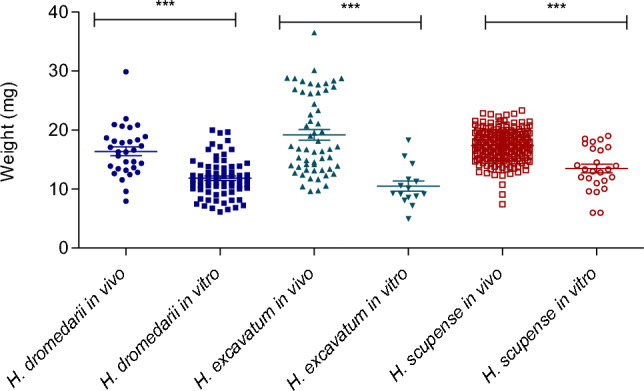
Variation of weight of *Hyalomma* nymphs fed in vivo and in vitro*.* Asterisks indicate level of significance using Mann Whitney test *P* < 0.0001 (***).

As for the females, the weight of engorged nymphs fed on animals was significantly higher than that of nymphs fed in vitro for all the three *Hyalomma* species (One-way ANOVA followed by Tukey test and confirmed by Mann Whitney test, *P* < 0.0001) (Fig. 6).

**Figure 7:**
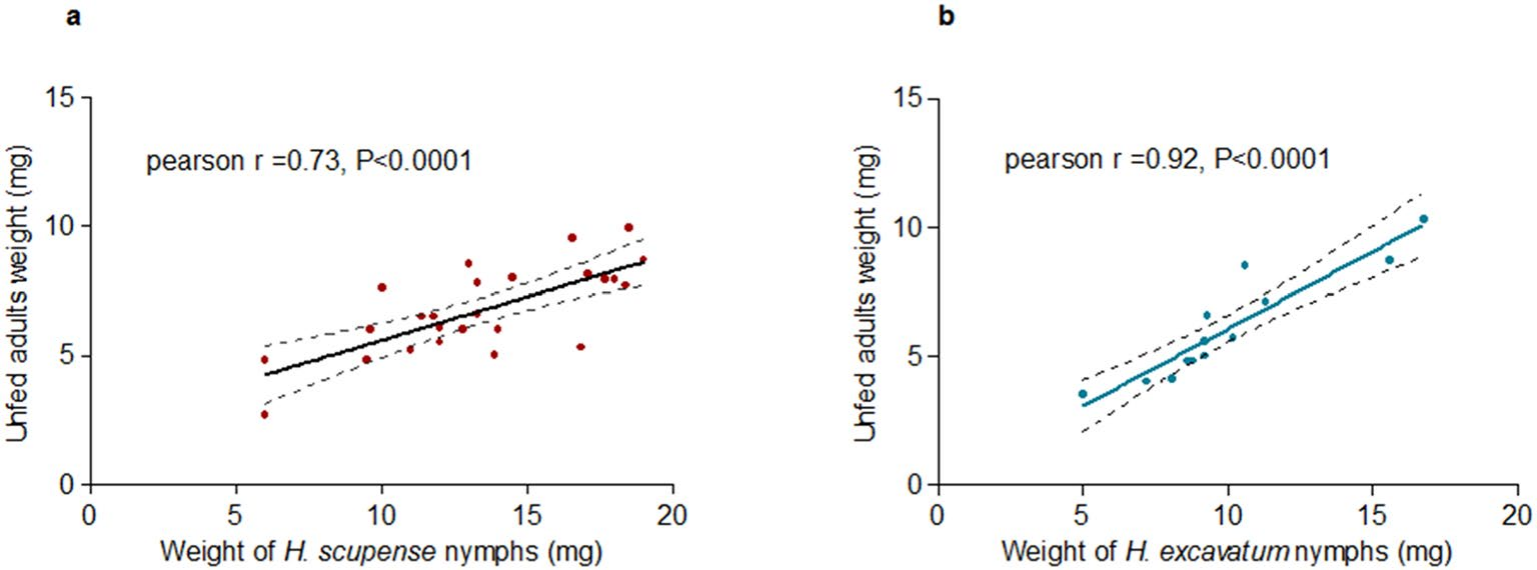
Correlation between the weight of engorged *H. scupense* (**a**) and *H. excavatum* (**b**) fed in vitro and the weight of unfed adults after molting.

There was a significant positive correlation between engorged *H. scupense* nymphs in vitro and the weight of their counterpart unfed adults (Pearson *r* = 0.73, *P* < 0.0001) (Fig. 7a). The same but stronger correlation was observed for unfed adult *H. excavatum* fed in vitro (Pearson *r* = 0.92, *P* < 0.0001) (Fig. 7b).

**Figure 8:**
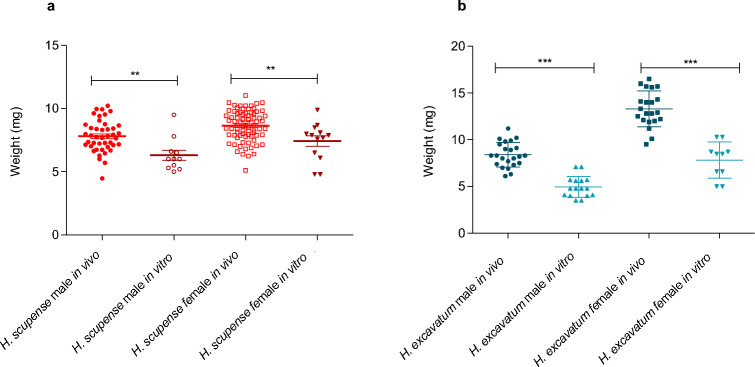
Difference between weight of unfed adults of *Hyalomma scupense* (**a**) and *Hyalomma excavatum* (**b**) obtained from nymphs fed in vivo and those fed in vitro. Asterisks indicate level of significance using Mann Whitney test (*P* < 0.01 (**) and *P* < 0.0001 (***).

The weight of unfed *H. scupense* adults obtained from in vivo feeding was significantly higher than those fed in vitro (One-way ANOVA and Tukey test, *P* < 0.0001) (Fig. 8a). The same trend was also reported for unfed *H. excavatum* adults (Mann Whitney test, *P* < 0.0001) (Fig. 8b). For both species fed in the two feeding conditions, unfed females had higher weight than unfed males (One-way ANOVA and Tukey test, *P* < 0.0001) except for *H. scupense* fed in vitro where the difference was not significant (Mann Whitney test, *P* = 0.06).

## Discussion

The use of artificial feeding systems to feed ticks in a laboratory setting instead of relying on vertebrate hosts has been used to study various aspects of tick biology, including host-tick interactions, tick feeding behaviour, and the transmission of tick-borne pathogens^24–26^. Feeding all life stages on membranes was previously reported for the three-host ticks *A. hebraeum*^15^, *A. variegatum*^27^ and *I. ricinus*^16^.

A number of studies describing the in vitro feeding of *Hyalomma* ticks have been previously published. This includes *H. dromedarii*, *H. anatolicum*^17,18^, *H. lusitanicum*^28^, *H. excavatum* and *H. marginatum*^29^. However, these studies were limited to the nymphal or adult stage. In this paper, we present the first successful in vitro feeding of all life stages of *H. dromedarii, H. scupense* and *H. excavatum* on silicone membranes*.*

### Feeding of *Hyalomma* adults

The highest engorgement rate was observed for adult *H. scupense* (76%) followed by *H. excavatum* (70%) and *H. dromedarii* (34.4%) with a mean feeding duration of 9, 7.6 and 11.5 days. These results were comparable to previous reports on the artificial feeding of *H. lusitanicum* which had an engorgement rate of 40.2% in approximately 11 days^28^. Similar results were previously reported by Tajeri and Razmi, (2011) for *H. dromedarii* (55%) and *H. anatolicum* (75%) with a feeding duration that varied between 11 and 16 days. The mean feeding duration found in this study for *H. dromedarii* (9.1 days) and *H. excavatum* (7.6 days) was shorter than the feeding durations of their counterparts fed on rabbits, which lasted 10.4 ± 1.8 days for *H. dromedarii* and 9.6 ± 2.5 days for *H. excavatum*. This is surprising as in vitro feeding usually lasts longer than the feeding on animals^16,30,31^. This could perhaps be attributed to the final feeding phase, which might have been quicker in vitro due to mechanical limitations caused by the confined space of the feeding units in combination with ticks clustering on the membrane in the feeding units, forcing the ticks detach earlier^25,31^ or perhaps a feeding stimulant factor(s) that was missing in the in vitro system. It would be interesting to observe how these ticks would behave in feeding units with a larger diameter or lower tick density.

A recent publication on the in vitro feeding *H. excavatum* adults*,* which originated from the same tick colony as used for this study, and *H. marginatum* showed much lower engorgement rates of 8.3 and 7.9%, respectively, with a longer feeding period of over 20 days^29^. Several factors might have led to this variation in feeding success such as tick fitness at time of feeding, feeding preferences and behaviour, membrane composition and thickness and specific environmental conditions such as temperature, relative humidity, CO_2_ levels, attachment stimuli, and dark/light condition^22,32^.

It has previously been suggested for *H. lusitanicum* that season could affect the tick feeding success (31.6%)^28^. Although it was limited to two experiments only, we did make similar observations for adult *H. scupense*, where we observed a higher engorgement rate in summer (June) (76%) compared to ticks fed in winter (January) (25%). This corroborates with the *H. scupense* adult activity in North Africa as these are mainly found on animals in summer^5,33^. However, more extensive experiments should be conducted to confirm whether the in vitro feeding success of *H. scupense* is truly season dependent.

Another important factor that can influence tick feeding efficacy is the feeding system itself. In a feeding system recently used for *R. appendiculatus*, the chambers were inverted (upside down) based on the natural tendency of ticks to crawl up towards a host^34,35^. Such an inverted system where the blood is placed above the membrane might also be useful for studies working on the artificial infection of ticks with intra-erythrocytic pathogens such as *Babesia* and *Theileria* spp.^32^.

In the present study, the highest mean detachment weight was recorded for *H. excavatum* (539.8 ± 167 mg) followed by *H. dromedarii* (305 ± 102 mg) and *H. scupense* S (298.01 ± 65.4 mg). The average weight of *H. excavatum* in this study is higher than the weight previously reported for *H. excavatum* fed in vitro (260.6 mg), where tick feeding did not take place inside an incubator but in a water bath to maintain a constant blood temperature. In general, the average weight of the engorged *Hyalomma* females was significantly higher for ticks fed in vivo than those fed in vitro (*P* < 0.0001), also leading to higher egg mass production. This finding is in line with previous report for *H. lusitanicum* fed in vivo^36^ and in vitro^28^ where the average weight were 543 mg and 274.65 mg, respectively. Higher average weights were also recorded for *I. ricinus* fed in vivo (231 ± 72.3 mg) compared to the ones fed in vitro (136 ± 44.9 mg)^16^. The lower engorgement weights of all fed life stages, resulting in smaller ticks and decreased egg production, is a consistent finding in the in vitro feeding of ixodid ticks when compared to feeding on natural hosts. This, in combination with the necessity to use antibiotics with potential negative effects on tick endosymbionts and tick fecundity remains a major stumbling block for the complete maintenance of tick laboratory colonies using artificial feeding alone. It will be essential to conduct long-term studies to examine whether *Hyalomma* tick colonies could also be maintained by in vitro feeding over several generations without detrimental side effects on the tick biology and physiology.

Despite the lower detachment weight, the percentage of engorged females that laid eggs was 50, 57.1 and 63.1% for *H. dromedarii, H. excavatum* and *H. scupense*, respectively. This was lower than their equivalents fed on animals, where 100%, 50% and 92.3% of the females laid eggs. The low oviposition rates observed in artificial feeding in comparison to in vivo feeding might be due to the elimination of endosymbionts or antibiotic toxicity. In general, the mean REI was around 50% for all three species under both feeding conditions (Table 1), except for *H. dromedarii* fed in vitro where the mean RE was 60%. This is higher than its counterparts fed on rabbit (49.5%) but in line with previous reports on *H. dromedarii* where high REI of up to 72% were reported^4^. This variation could be explained by host-differences, it was for instance shown that *H. anatolicum* had a higher REI when fed on a rabbit compared to sheep and goats^37^, but also by the tick strain, quantity of blood ingested, humidity, temperature and possible disturbance during oviposition^38^.

### Feeding of immature stages

The engorgement rate of larvae in vitro was 42.8%, 56.8% and 55.7% for *H. dromedarii*, *H. excavatum* and *H. scupense*, respectively. These proportions are in line with those obtained for *I. ricinus* fed in vitro (55%) and in vivo (41%)^16^. An engorgement rate of 71% was obtained for *R. australis* larvae using a different feeding system, where most of the larvae attached after 48 h which is similar to the observations made in this study^39^. The encouraging engorgement rate in our study may be associated to the presence of attachment stimuli (hair extract and rabbit hair). The thickness and type of membrane appear to be crucial factors for the larval and nymphal feeding success. As *Hyalomma* juvenile ticks have a relatively short hypostome, varying from 54 to 119 μm for the larvae and 156–268 μm for the nymphs^9,29^, they require membranes thin enough to pierce through and feed while avoiding leakage. This problem might explain why immatures stages of other *Hyalomma* species could reportedly not feed or fed with low attachment rates^28,29^. In addition, the age of the ticks and the season in which they are fed were also suggested to influence the efficacy and the success of the tick feeding^16^.

*Hyalomma anatolicum* nymphs were previously fed on mouse skin membrane with an engorgement rate reached 89% with a feeding duration varied from 7 to 8 days in a water bath set to 37 °C^17^. The high engorgement rate reported in that study is related to the use of animal skins, which do not require external stimuli to simulate attachment and more closely mimic natural feeding conditions. However, the use of animal skin may cause increased contamination when maintained at 37 °C, especially when used for one- or two-host tick species which have a longer feeding duration, as well as bioethical concerns^21,32^.

In our study, the feeding-molting duration was between 9.3 ± 2.5 and 16.5 ± 3.5 days for *H. excavatum* and *H. scupense*, respectively. The longer feeding duration, especially when the tick are not attached synchronously (Fig. 3) as previously reported^18,28^, increase the risk of contamination inside the feeding unit. For *H. scupense*, partially engorged larvae were moved manually to another feeding unit where not all of them reattached, which explains why only 55.7% of the initially placed larvae engorged. The engorged larvae molted to nymphs on the membrane (Supplementary Fig. 1). The freshly molted nymphs were transferred to a new feeding unit where the engorgement rate was only 36.8% (Table 2). The fungal contamination inside the chamber was the main factor that affected the engorgement rate of the immature stage of both two-host ticks (*H. excavatum* and *H. scupense)*. To prevent this from happening, we tried using nystatin cream to clean the inner side of the membrane and the addition of Whatman filter paper and bags with silica gel to absorb the humidity inside the feeding unit. These precautions appeared to help in delaying the contamination until most of the nymphs detached, but did not completely prevent contamination from occurring. In general, the transfer of immature ticks to clean feeding units and other precautions to limit contamination makes the feeding laborious, and future studies may focus on optimising these steps in order to improve the engorgement rate for one- and two-host tick species in vitro.

The average weight of nymphs fed in vitro was 12.7 ± 3.1, 10.4 ± 2.8 and 13.4 ± 3.6 mg for *H. dromedarii*, *H. excavatum* and *H. scupense*, respectively. As for the females, the nymphal weight was significantly higher for ticks fed on animals compared to in vitro fed ticks and showed a significant difference between the three *Hyalomma* species (Mann Whitney test, *P* < 0.0001). Lower detachment weights of all stages fed in vitro were also recorded for *I. ricinus*^16^. Although the weights were lower, larvae and nymphs fed in vitro had weights that allowed them to successfully molt to the next stage. We also observed that incomplete feeding of nymphs lead to their death or resulted in smaller adults, similar to previous reports^40,41^. We note a significant positive correlation between engorged *H. scupense* and *H. excavatum* nymphs fed in vitro and the weight of their counterpart unfed adults where the weight of unfed female is higher than males. These observations were in line with previous reports on *I. ricinus*, *I. scapularis* and *Dermacentor variabilis*^16,42^ which showed that weight of nymphs that become female imbibe more blood than those became male.

The reported in vitro feeding method was successfully used to feed all *H. excavatum*, *H. dromedarii* and *H. scupense* life stages. While there are some similarities in their response to in vitro feeding, some variations specific to the species existed which require more investigation and understanding these differences will improve our knowledge of tick biology, tick-borne disease transmission, and improve the development of control measures. The ATFS could also contribute to the 3Rs by reducing the use of laboratory animals required to maintain tick colonies, although the impact of in vitro feeding on tick microbiota due to the use of antibiotics requires further investigation.

## Materials and methods

### Maintenance of tick colonies

Colonies of *Hyalomma scupense* and *H. dromedarii* used in this study were originally obtained from the National School of Veterinary Medicine of Sidi Thabet, Tunisia and the *H. excavatum* ticks originated from a colony of the Aydın Adnan Menderes University, Türkiye. The colonies were maintained at the Institute of Parasitology and Tropical Veterinary Medicine of the Freie Universität Berlin, whereby the juvenile life stages were fed on gerbils or the ears of rabbits. The adults were fed on the ears of rabbits (for *H. dromedarii* and *H. excavatum*) or calves (for *H. scupense*) in linen bags. Ethical approval for the feeding of ticks on experimental animals was granted by the Landesamt für Gesundheit und Soziales, Berlin, Germany (LAGeSo) under Registration Number H0387/17 and 0144/22.

Collected ticks were stored in an exsiccator at 27 °C or at room temperature with approx. 90% relative humidity (RH). Data from the feeding of ticks on experimental animals for tick colony maintenance were recorded for comparison purpose with in vitro feeding results.

### In vitro feeding of *Hyalomma* ticks

The methods for the in vitro feeding of all *Hyalomma* life stages were adapted from previously published protocols^16,28^. Ticks were fed on a silicone membrane based on a matrix of goldbeater’s skin (for larvae and nymphs) or lens cleaning paper (for adults) with thicknesses of 40–50 μm for larvae, 50–70 μm for nymphs and 80–120 μm for adults. The feeding units were placed in a 6-, or 12-well cell culture plate, depending on the unit's diameter and placed in an incubator (ICH110C, Memmert, Schwabach, Germany) at 27 °C, 70% RH and 5% CO_2_ in complete darkness. The culture plates were placed on a heating plate (hot plate 062, Labotect, Göttingen, Germany) set at 42 °C to keep the blood warm. Bovine hair extract and rabbit hair was added as an attachment stimulus on top of the silicone membranes approx. 45 min before ticks were introduced into the units. Bovine hair extract was prepared as described by Militzer et al.^16^. Briefly, approximately 50 g of fresh bovine hair was immersed three times for two hours each in different ratios of chloroform–methanol mixture (2:1, 1:1 and 1:2). The first two immersions took place at room temperature, the final step at 45 °C. Extracts from each immersion step were collected and combined, vacuum filtered and concentrated by roto-evaporation. Finally, the bovine hair extract was dissolved in a 1:2 chloroform–methanol mixture, aliquoted to small working solutions and stored at − 20 °C until use. One hundred μl of bovine hair extract was used for feeding units with a diameter of 30 mm and 50 μL for feeding units with a diameter of 20 mm. The feeding units were closed using a mite plug made from polyurethane foam (K-TK e.K., Retzstadt, Germany) to allow for gas exchange while preventing the ticks from escaping. Defibrinated aseptic bovine blood was purchased from Xebios Diagnostic (Düsseldorf, Germany) and supplemented with 0.1 M adenosine triphosphate (ATP, Carl Roth, Karlsruhe, Germany), vitamin B^43^, 2 mg/mL glucose and 5 μg/mL gentamicin prior to each blood change, which took place at 14:10 h intervals. The vitamin B was added based on previous observations by Militzer et al.^16^ suggesting that this may result in better engorgement and fecundity rates. To avoid contamination of the feeding unit, the underside of the membrane was washed with 1% nystatin diluted in PBS, followed by 0.9% NaCl at each blood change.

Once ticks were attached, the rabbit hair used to stimulate attachment was removed, as well as any dead ticks and faeces produced by the ticks during feeding to keep the feeding unit as clean as possible. The inner side of the feeding unit was initially cleaned with 1% nystatin in PBS when fungal contamination was observed. As this increased the humidity inside the feeding units and caused droplets to form that wetted the membrane and made it unsuitable for ticks to feed, we subsequently switched to the treatment of fungal contamination with a nystatin cream (Nystalocal, Pierre Fabre, Freiburg) and also added a Whatman filter paper to cover the attached ticks and bags with silica gel to absorb excess humidity to the feeding unit. In cases where this treatment was not successful, ticks were manually removed, washed in 0.9% NaCl and dried with tissue paper before being transferred to a newly prepared feeding unit. In each ø 30 mm feeding unit, ten male and ten to 12 female ticks were placed. For the in vitro feeding of the immature stages, a maximum of approx. 75 nymphs or 150 larvae were placed in each ø 30 mm unit. One group was fed for each life stage, with the exception of the *H. scupense* adults for which one group was fed in summer and one in winter.

### Biological parameters

The rates of attached and engorged ticks were recorded for all life stages, as well as additional parameters such as detachment weight, molting rates and reproductive parameters including oviposition duration, weight of egg mass and oviposition-hatching duration. The number of eggs for each species was estimated by dividing the weight of the egg batch with the weight of a single egg, whereby the average egg weight was 0.058 ± 0.009 mg for *H. dromedarii*, 0.057 ± 0.001 mg for *H. scupense* and 0.053 ± 0.008 mg for *H. excavatum* as measured on an analytic scale. The percentage of hatched larvae was estimated visually under binocular stereoscope by two persons^44^. The following parameters were calculated:

$$\begin{aligned} & {\text{Engorgement}}\;{\text{rate}}\;\left( \% \right) = \left( {{\text{number}}\;{\text{of}}\;{\text{engorged}}\;{\text{females}}/{\text{numbers}}\;{\text{of}}\;{\text{initially}}\;{\text{placed}}\;{\text{ticks}}} \right)*{1}00 \\ & {\text{Reproductive}}\;{\text{efficiency}}\;{\text{Index}}\;\left( {{\text{REI}}} \right)\;\left( \% \right) = ({\text{egg}}\;{\text{mass}}/{\text{female}}\;{\text{engorgement}}\;{\text{weight}})*{1}00 \\ \end{aligned}$$

### Statistical analyses

Statistical analyses and graphs were generated using GraphPad Prism 5 windows (GraphPad Software, San Diego California, United States). Chi-square test was used to compare the feeding proportions between the three species and Z-test was used to compare two proportions using an online version of epitools (http://epitools.ausvet.com.au). One-way ANOVA followed by Tukey-test was used to assess the difference in feeding parameters such as feeding duration, weight, pre-oviposition and oviposition-hatching duration between the tick species in vivo and in vitro (between more than two groups). The Mann–Whitney test was used to analyse the variation of some parameters between two groups and to confirm the results of one-way ANOVA test. Pearson correlation and linear regression were performed to assess the relationship between the following parameters: female weight and egg mass produced, engorged nymphs' weight and their corresponding unfed adult weight. For all test used, a 5% threshold value was considered as statistically significant.

### Supplementary Information

Supplementary Information.

## Data Availability

All data generated or analysed during this study are included in this published article [and its supplementary information files].
