# Supplementary material for: In vitro feeding of all life stages of two-host Hyalomma excavatum and Hyalomma scupense and three-host Hyalomma dromedarii ticks
Source: Sci Rep. 2024 Jan 3;14:444. doi: 10.1038/s41598-023-51052-w (PMC10764919; doi:10.1038/s41598-023-51052-w)
Supplement: Supplementary file 1 — Supplementary Information. [file 41598_2023_51052_MOESM1_ESM.docx]

**Supplementary table 1:** Vitamin B component and concentrations

| **Vitamin B component** | **Final concentration (in blood meal)** | **Solvent** |
| --- | --- | --- |
| thiamine | 100µg/mL | 0.9% NaCl |
| riboflavin | 20µg/mL | 0.9% NaCl |
| nicotinic acid | 100µg/mL | 0.9% NaCl |
| panthotenic acid | 100µg/mL | 0.9% NaCl |
| pyridoxine | 100µg/mL | 0.9% NaCl |
| biotin | 1µg/mL | 0.9% NaCl |
| folic acid | 30µg/mL | 1N NaHCO3 |
| cyanocobalamin | 1µg/mL | 0.9% NaCl |
| chlorine chloride | 185µg/mL | 0.9% NaCl |
| myo-inositol | 118µg/mL | 0.9% NaCl |


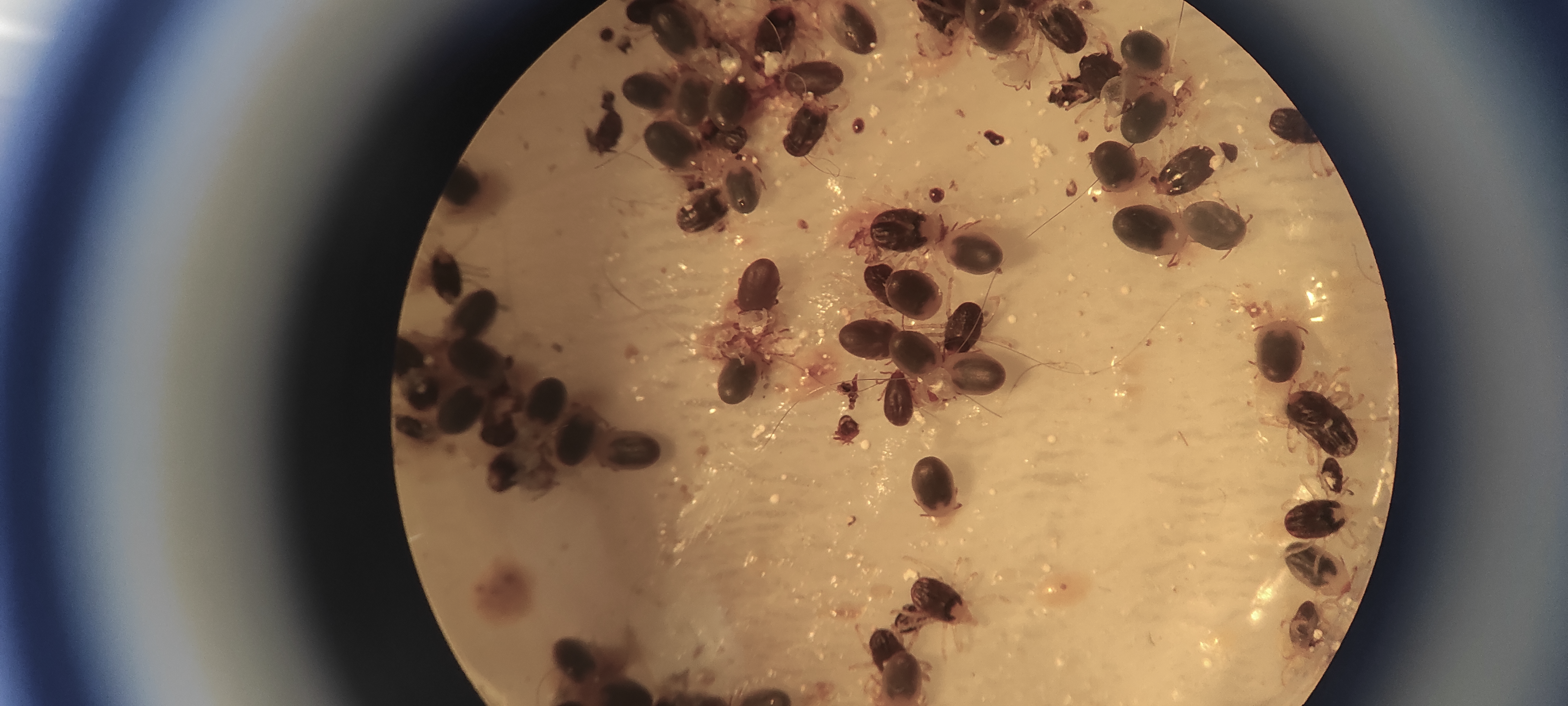


**Supplementary figure 1.** Molting of *Hyalomma scupense* engorged larvae on the membrane
